# Hydration Status in Men Working in Different Thermal Environments: A Pilot Study

**DOI:** 10.3390/ijerph19095627

**Published:** 2022-05-05

**Authors:** Joanna Orysiak, Magdalena Młynarczyk, Paweł Tomaszewski

**Affiliations:** 1 Department of Ergonomics, Central Institute for Labour Protection—National Research Institute, Czerniakowska St. 16, 00-701 Warsaw, Poland; m.mlynarczyk@ciop.pl; 2 Department of Tourism and Recreation, Józef Piłsudski University of Physical Education, Marymoncka St. 34, 00-968 Warsaw, Poland; pawel.tomaszewski@awf.edu.pl

**Keywords:** hypohydration, dehydration, urinary indices, osmolality, urine-specific gravity, foresters, workers

## Abstract

The aim of this study was to determine the effects of different seasons of the year and the time of day (before work vs. after work) on hydration status in men. The study involved sixty foresters who spent most of the work outdoors. During three seasons of the year (summer, autumn, and winter), indices of hydration status (body mass (BM) and percentage change of BM, total body water (TBW) and percentage change of TBW, serum osmolality (S_osm_) and percentage change of S_osm_, urine osmolality, urine-specific gravity (USG), urine color, and thirst) were determined before work on the first day (time point 1 used as baseline), immediately after work on the first day (time point 2), and before work on the following day (time point 3). USG decreased at time point 2 compared to time point 1 (*p* < 0.001) and time point 3 (*p* = 0.03). At time point 2 (*p* = 0.002) in winter and time point 3 in autumn (*p* = 0.049), serum osmolality was higher than in summer. In conclusion, the differences in hydration status depended on the time of day and season. A large percentage of foresters come to work inadequately hydrated, especially in colder seasons compared to summer.

## 1. Introduction

In many professions, work is performed in unfavorable microclimate conditions, at low or high temperatures. Using physiological and behavioral mechanisms, the human body can maintain a constant internal/core body temperature (approx. 37 ± 1 °C), despite the varied influence of the thermal environment [1]. However, when heat balance is disturbed, it affects both physical performance and human health [1]. When working in a hot environment, workers could be exposed to heat stroke, heat exhaustion, heat cramps, heat rashes, injuries (for example, chronic kidney injuries), or dehydration [2,3,4]. In turn, working in a cold environment increases the risk of hypothermia, frostbite, and non-freezing injuries such as immersion foot, chilblains, or cracked skin [5]. People working in these thermal environments are exposed to dehydration, even at low temperatures, when using, for example, improper protective clothing [6,7]. 

There is no universally recognized definition for dehydration. However, in clinical terms, it was defined as a deficiency of total body water [8]. Previous studies have suggested that dehydration is a process of losing body water, while hypohydration is the state in which body water deficit occurs [9]. Dehydration is one of the factors that can affect the health, productivity, and safety of workers at work [10,11,12,13]. It was shown that even slight dehydration of 1% may disturb the mechanisms of thermoregulation [10,11,12,13]. In addition, greater dehydration affects decision-making and the ability to perform physical effort, which in turn reduces safety and may lead to an increase in the number of accidents at work [10,11,12,13,14].

There are several methods to assess hydration status in humans, the most common being based on the evaluation of body mass changes (%BM), total body water changes (%TBW), thirst, plasma/serum osmolality (S_osm_), or urine osmolality (U_osm_), urine color (U_col_), and urine-specific gravity (USG) [8,13,15,16,17]. Each method of assessing the hydration status has its advantages and disadvantages. Until now, serum/plasma osmolality was considered the gold standard for hydration status assessment [8,18,19], although its usefulness is increasingly being questioned [20,21]. In studies conducted among physical workers, the hydration status is often estimated based on urine-specific gravity [14,22,23,24,25,26,27,28,29,30]. However, this method also has disadvantages, including the need to use specialized equipment and employing highly experienced operators [17]. There are currently no clear recommendations on which indicators of hydration status should be monitored in humans, and no single marker is considered definitive [17]. According to Cheuvront et al. [19], a useful marker to determine static (one-time) dehydration is plasma osmolality, while dynamic (monitoring over time) dehydration can be assessed with plasma osmolality (P_osm_), USG, and body mass measurements. Based on the recommendations of Kenefick and Sawka [11], it is best to record morning body mass, urine color, and thirst. 

It was suggested that maintaining good hydration status may be a simple and economical strategy to prevent occupational heat stress [3,31]. As some workers start working already in a state of inadequate hydration, and their hydration status may deteriorate during work [11], it is important to pay attention to the hydration status of workers, especially in different environmental conditions. 

The aim of this study was to determine the effects of different seasons of the year and the time of day (before work vs. after work) on hydration status in men. The study examined foresters (who spend most of their work outdoors) to illustrate the work in various environmental conditions.

## 2. Materials and Methods

### 2.1. Participants

The study included men (*n* = 60) who worked as foresters. This work involves outdoor activity in various thermal conditions. 

Foresters were selected at their workplaces. The information on the selection for research and inclusion criteria (gender, age, health condition) was sent to each workplace. Then, before the tests, the researchers made an additional selection, taking into account the exclusion criteria. 

Participants were excluded from the study if they met any of the following criteria:

- A pacemaker or other electronic devices implanted under the skin;
- Metal elements under the skin (e.g., plates, screws, etc.);
- Symptoms of an infection on the day of the examination;
- Chronic diseases, e.g., diseases of the urinary system, metabolic diseases, cardiovascular diseases;
- Use of medications, e.g., diuretics [32];
- Over 45 years of age.

Ultimately, 60 participants were qualified for the study.

### 2.2. Study Design

A cross-sectional observational design was employed to examine a group of voluntary foresters during three seasons of the year (summer, autumn, and winter). In each examination, three urine and blood samples were obtained from study participants, and body mass and total body water were measured before work on the first day (time point 1 was used as a baseline), immediately after work on the first day (time point 2), and before work on the following day (time point 3). The following hydration status indices were assessed during each collection of biological material:

- Urine color (U_col_);
- Urine osmolality (U_osm_);
- Urine-specific gravity (USG);
- Serum osmolality (S_osm_);
- Body mass (BM);
- Total body water (TBW);
- Thirst (questionnaire).

In order to maintain similar conditions during all test periods/seasons, the test day was always preceded by a working day, not a day off from work. 

Each volunteer was tested only during one season of the year. Each worker was measured over one shift. The shift at work lasted at least 6 h. Foresters were instructed not to change their drinking and eating habits, i.e., to eat and drink in the usual amounts both before and during the research. Participants were not previously educated on the correct/proper fluid intake. 

The study was approved by a local ethics committee, and written informed consent was obtained from participants. The study was conducted in accordance with the principles of the Helsinki Declaration.

### 2.3. Measured Parameters

The research was carried out by qualified personnel with many years of experience in collecting biological material (including blood) and conducting body mass and composition measurements. During the organizational meeting, the participants were instructed how to correctly collect urine for testing.

#### 2.3.1. Workplace Microclimate

On the test day, the microclimate parameters at the workplace (air temperature, relative humidity) were measured using the HygroLog NT modular data recording system (HL-NT2-DP, IP65, Rotronic AG, Bassersdorf, Switzerland). The information about the average weekly temperature during working hours in the vicinity of the research site was obtained from publicly available data from the Institute of Meteorology and Water Management—National Research Institute [33].

#### 2.3.2. Thirst

Thirst was assessed using the questionnaire item “Do you feel thirsty now?” with “Yes” or “No” answers. 

#### 2.3.3. Anthropometric Measurements (Total Body Water and Body Mass)

Measurements of body mass and body composition (including total body water) were made using an Inbody 270 body composition analyzer (Biospace, CA, USA) in the morning in the fasting state before work (time points 1 and 3) and after work (time point 2) (without waiting a min. 2 h after food and fluid intake). Height was measured to the nearest 0.1 cm using a Seca 213 stadiometer (Hamburg, Germany). According to World Health Organization (WHO), a body mass index (BMI) of 18.5–24.9 is considered normal weight, over 25 is overweight, and over 30 is obese [34]. 

#### 2.3.4. Urine Indices of Hydration Status

Urine for the assessment of hydration status was collected in the morning, in the fasting state before work (urine was collected from the middle stream from the first morning voiding), and after work (when urine was in the bladder for approx. 2 h before sample collection). Urine was delivered to the laboratory immediately after collection. Urine-specific gravity was determined at the workplace, while urine osmolality was determined from frozen samples (at −20 °C) in a diagnostic laboratory. Urine osmolality was determined by a freezing-point osmometer (cryoscopic method) (Marcel OS3000 osmometer, Poznań, Poland).

Urine-specific gravity was evaluated at the workplace using a PAL-10S Atago refractometer (Tokyo, Japan). Both urine-specific gravity and urine osmolality were determined in duplicate. 

Urine color was assessed using the 8-point scale described by Armstrong et al. [35] by two independent persons who kept urine containers close to the scale in a well-lit room [35].

#### 2.3.5. Serum Hydration Indices

Blood for serum osmolality testing was taken from the fingertip (capillary blood) and then centrifuged at 3000 rpm and frozen at −20 °C for further determinations in a diagnostic laboratory. Serum osmolality was determined by the cryoscopic method using a Marcel OS3000 osmometer (Poznań, Poland).

### 2.4. Measured Hydration Indices

#### 2.4.1. Estimating Percentage Changes of Indices (Body Mass, Total Body Water, Serum Osmolality)

The percentage loss of specific indices (%BM, %TBW, %S_osm_) relative to time point 2 or time point 3 was calculated as [25]:

(1) $$\%X=\frac{\left(X_{post}-X_{pre}\right)}{X_{pre}}\times 100\%$$

where:

%*X*—percentage change of body mass or percentage change of total body water or percentage change of serum osmolality;
*X_post_*—marker of hydration status after work (time point 2) or before work on the following day (time point 3);
*X_pre_*—marker of hydration status before work on the first day (time point 1).

#### 2.4.2. Criteria for Hydration Status Indices

There are many criteria for assessing hydration status. In the present study, the criteria adopted for dehydration are presented in Table 1.

### 2.5. Statistical Analysis

Basic statistical measures were used to describe the results: arithmetic means, standard deviations (SD), and percentages. The assumption of normality of the distributions of analyzed variables was tested using the Shapiro–Wilk test, whereas homogeneity of variance was verified using the Levene test. Because the assumptions of parametric tests were not met and the group sizes were relatively small, differences between time points and the percentage changes from baseline were assessed using the non-parametric procedures of the Freidman test and Wilcoxon test, respectively. The Bonferroni correction was used in the case of multiple Wilcoxon test comparisons. Consequently, the differences for BMI [34] categories or between seasons were tested using the Kruskal–Wallis test and followed by a multiple median comparison test (post hoc). For categorical variables, the chi-squared test was used, with Yate’s correction (2 × 2 tables) or maximum likelihood variant (larger tables). The relationships between variables were tested using Spearman’s rank correlation. Analyses were performed using STATISTICA 13 (TIBCO Software, Palo Alto, CA, USA). The level of *p* < 0.05 was set to evaluate the significance of the effects.

## 3. Results

The results for the analysis depending on the time of day or season of the year are presented below.

### 3.1. General Information

#### 3.1.1. Participants

The characteristics of the study participants are presented in Table 2. None of the variables showed significant differences between the seasons of the year. Twenty-nine participants were overweight, and seventeen were obese. Analysis with the division into BMI showed a significant difference between workers with BMI < 25 and above 25 only in the case of total body water (Kruskal–Wallis H = 13.9; *p* < 0.001) and body fat percentage (Kruskal–Wallis H = 38.1; *p* < 0.001). Due to the lack of effect of BMI on the indices of hydration status (thirst, percentage change in total body water, percentage change in body mass, urine color, urine-specific gravity, urine osmolality, and serum osmolality), the authors decided to perform a combined analysis without distinguishing between the BMI values.

#### 3.1.2. Environmental Conditions

The microclimate parameters controlled at the workplace in the middle of the shift were 20.5 ± 0.0 °C and 57.7 ± 0.1% in summer, 16.8 ± 0.0 °C and 51.2 ± 0.0% in autumn, and 4.3 ± 0.0 °C, and 68.5 ± 0.1% in winter for air temperature and relative humidity, respectively. The average weekly temperature during working hours in the vicinity of the research site was 22.3 ± 3.2 °C in summer, 10.9 ± 4.0 °C in autumn, and 0.5 ± 2.9 °C in winter.

### 3.2. Hydration Markers in All Participants at Time Points

The results of urine markers assessment at each time point (time point 1—before work used as a baseline, time point 2—after work, and time point 3—before work on the following day) for all participants are shown in Table 3.

#### 3.2.1. Thirst

There were no differences in the prevalence of self-reported thirst between the three time points. However, it should be noted that about half of the participants felt thirsty at time points 1 and 3, and a third of them felt thirsty at time point 2 (Table 3).

#### 3.2.2. Body Mass (BM)

Significant differences in body mass between time points were found (Freidman chi-squared = 22.4; *p* < 0.001). After work (time point 2), body mass was significantly higher compared to baseline (time point 1; *p* < 0.001) and before work on the following day (time point 3; *p* < 0.001), with a mean increment of around 0.2 kg (Table 3).

**Figure 1:**
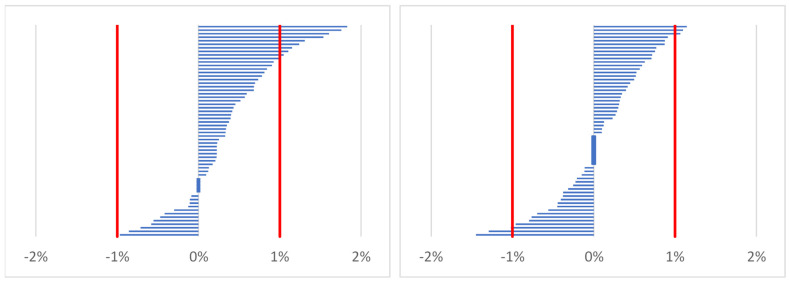
Distribution of changes in body mass for time point 2 (after work; on the left) and time point 3 (before work on the following day; on the right) vs. time point 1 (before work/baseline) in all participants (*n* = 60).

For the percentage change in body mass (weight gain > 1%), a greater increase in body mass was observed at time point 2 compared to time point 3 (*p* < 0.001). As shown in Figure 1, none of the participants lost more than 1% of their body mass during work compared to before work on the first day (between time points 2 and 1), but before work on the following day, three foresters lost more than 1% of body mass compared to before work on the first day (between time points 3 and 1). 

#### 3.2.3. Total Body Water (TBW)

**Figure 2:**
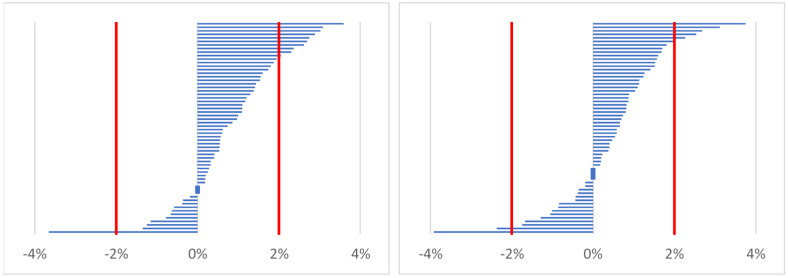
Distribution of changes in total body water for time point 2 (after work; on the left) and time point 3 (before work on the following day; on the right) vs. time point 1 (before work/baseline) in all participants (*n* = 60).

The total body water differed significantly between the different time points (Freidman chi-squared = 26.5; *p* < 0.001). On the first day (baseline, time point 1), the values of total body water were lower than after work (time point 2) (*p* < 0.001) and before work on the following day (time point 3) (*p* = 0.015). No significant differences were observed in the percentage change in total body water. As shown in Figure 2, a decrease of less than 2% compared to baseline was observed in only one participant after work (between time points 2 and 1) and in two participants on the following morning (between time points 3 and 1). In turn, an increase in the total body water of above 2% was recorded in 10 foresters at time point 2 and in 5 participants at time point 3 (Figure 2).

#### 3.2.4. Urine Color (U_col_)

Irrespective of the time point (Freidman chi-squared = 5.82; *p* = 0.055), the average urine color was 4.0, which is considered an upper range of fairly hydrated. However, based on the assessment of urine color, 30–43% of the foresters were described as improperly hydrated (they obtained scores of 5 and above on the urine color scale) (Table 3). 

#### 3.2.5. Urine-Specific Gravity (USG)

Analysis of urine-specific gravity indicated a significant effect of time (Freidman chi-squared = 15.4; *p* < 0.001), with a significant decrease in USG after work (time point 2) compared to the values obtained before work on the first day (time point 1; *p* < 0.001) and before work on the following day (time point 3; *p* = 0.03) (Table 3). At least 50 percent of the workers were dehydrated during each time point (Table 3). Based on the more detailed criteria used by Biggs et al. [25], it was found that 22%, 3%, and 12% of foresters were extremely dehydrated (USG > 1.027), before work on the first day (time point 1), after work (time point 2) and before work on the following day (time point 3), respectively. Furthermore, 8%, 13%, and 3% of foresters at time points 1, 2, and 3, respectively, were extremely hydrated (USG < 1.012) (data not shown).

#### 3.2.6. Urine Osmolality (U_osm_)

There were no statistically significant differences in urine osmolality between the time points (Freidman chi-squared = 2.63; *p* = 0.27). As in the case of other urine indices, also for urine osmolality, more than half of the workers were dehydrated at all three time points (Table 3). At time point 1, only 35% of workers were adequately hydrated.

#### 3.2.7. Serum Osmolality (S_osm_)

**Figure 3:**
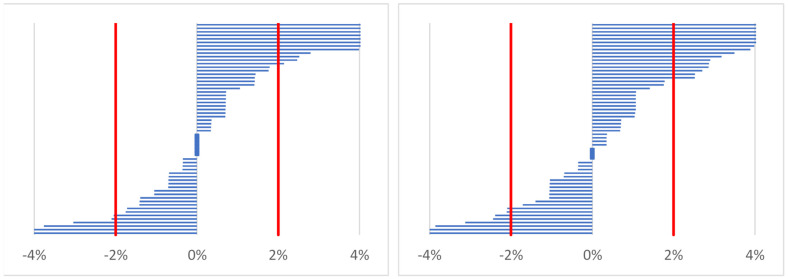
Distribution of changes in serum osmolality for time point 2 (after work; on the left) and time point 3 (before work on the following day; on the right) vs. time point 1 (before work/baseline) in all participants (*n* = 60).

No significant differences were found between serum osmolality (S_osm_) obtained at different time points (Freidman chi-squared = 4.61; *p* = 0.10). However, based on serum osmolality values equal to or above 290 mOsmol/kg H_2_O, 12% of participants were dehydrated at time points 1 and 2, while 17% were dehydrated at time point 3. Similarly, 20% (time point 2 versus time point 1) and 27% (time point 3 versus time point 1) had an increase in serum osmolality above 2%, which may indicate dehydration in these workers (Figure 3).

#### 3.2.8. Correlations between Urine and Serum Markers

Significant moderate relationships were found between the self-reported thirst at time points (correlation coefficients ranging from 0.354 to 0.504) and between body mass and TBW percentage changes after work (r = 0.417) and before work on the following day (r = 0.541). The most profound relationships were noted for urine characteristics, with urine color correlated with USG (r from 0.702 to 0.739) and U_osm_ (r from 0.558 to 0.654) and the correlations between USG and U_osm_ being the highest and ranging from 0.869 to 0.940. A significant but weak correlation was also found between USG and S_osm_ measurements at baseline (r = 0.306), whereas other relationships between urine and serum indices proved not to be significant.

### 3.3. The Impact of the Season of the Year on Hydration Markers

The results of hydration markers assessment depending on the season of the year (summer, autumn, winter) are shown in Table 4.

There were no differences in self-reported thirst between seasons of the year (Table 4) at respective timepoints (chi-squared from 0.34; *p* = 0.84 to 4.75; *p* = 0.09). The same was true for body mass (Kruskal–Wallis H < 1.73; *p* > 0.42), while for TBW, the only significant difference was found for time point 1 (Kruskal–Wallis H = 7.6; *p* = 0.022), with significantly higher (*p* = 0.02) values observed in winter compared to summer. There was also no significant effect of the season on urine indices of hydration status, with chi-squared ranging from 0.04 (*p* = 0,98) to 3.17 (*p* = 0.21). However, it should be emphasized that in all seasons of the year (different temperature conditions), the average values obtained for urine-specific gravity and urine osmolality were in most cases above 1.020 g/mL and 700 mOsmol/kg H_2_O. Moreover, the percentage of workers considered dehydrated varied depending on the indices. Based on urine color, 40–55% of workers in summer, 24–43% of workers in autumn, and 26–47% of workers in winter were classified as dehydrated. The percentage of workers who qualified as dehydrated based on urine-specific gravity ranged from 45 to 70% in summer, from 48 to 57% in autumn, and from 42 to 68% in winter. For urine osmolality, a similarly high percentage of workers considered dehydrated was observed (50–75% in summer, 52–62% in autumn, and 58–68% in winter). 

There were also no significant differences in the percentage change of body mass between the seasons of the year (Kruskal–Wallis H = 1.80; *p* = 0.41 and H = 0.26; *p* = 0.88 for time points 2 and 3 vs. time point 1, respectively) or total body water percentage change (Kruskal–Wallis H = 3.33; *p* = 0.19 and H = 3.11; *p* = 0.21). The changes did not exceed 0.35% for body mass and 0.63% for TBW. 

On the other hand, depending on the season, changes in the concentration of serum osmolality were revealed for time point 2 (Kruskal–Wallis H = 12.3; *p* = 0.002) and time point 3 (Kruskal–Wallis H = 6.27; *p* = 0.044) but not for time point 1 (Kruskal–Wallis H = 3.67; *p* = 0.16). At time point 2 in winter (*p* = 0.002) and time point 3 in autumn (*p* = 0.049), higher serum osmolality values were recorded compared to the respective time points for summer. There was also a statistically significant difference in the percentage of dehydrated workers based on serum osmolality for all time points (chi-squared from 9.11; *p* = 0.010 to 11.5; *p* = 0.003). In winter, the percentage of dehydrated participants was significantly higher at time points 1, 2, and 3 compared to the respective time points in summer and higher at time points 1 and 2 compared to autumn (Table 4). Moreover, the percentage of workers qualified as dehydrated based on the percentage changes in serum osmolality at time point 2 compared to time point 1 differed significantly (chi-squared = 9.02; *p* = 0.01), with a higher (*p* < 0.05) percentage observed in winter (42%) compared to autumn (14%) and summer (5%). A comparison of the percentage changes in serum osmolality at time point 3 with time point 1 (chi-squared = 9.05; *p* = 0.01) revealed a higher percentage of dehydrated workers in winter (42%) and autumn (33%) compared to summer (5%; *p* < 0.05).

### 3.4. Comparison of the Percentage of Workers Who Qualified as Dehydrated Depending on the Hydration Status Indices

Depending on the hydration status indices, dehydration in foresters after work (time point 2) ranged from 0% (for percentage change in body mass; no one met the criterion) to 61.7% (for urine osmolality). Similarly, before work on the following day (time point 3), the percentage of dehydrated foresters, depending on the hydration status indices used, ranged from 3.3% (for percentage change in total body water) to 53.3% for urine osmolality (Table 5). Evaluation of workers’ dehydration at time point 3 based on serum osmolality differs significantly from that based on urine indices of hydration status (Table 5). 

## 4. Discussion

In the present study, the differences in hydration status depended on the time of day (before or after work). Based on urine-specific gravity (USG), a large percentage of foresters came to work inadequately hydrated, and their hydration improved during their work (USG values decreased after work). Moreover, a higher concentration of serum osmolality was found in autumn and winter compared to summer, which is reflected in a higher percentage of dehydrated workers in colder seasons compared to summer. Moreover, the percentage of workers identified as dehydrated differed depending on the indices of hydration status.

Maintaining proper/optimal hydration has a positive effect on health and physical and mental fitness/performance. Therefore, it is important to monitor the hydration status [11,13]. However, hydration status assessment is difficult to interpret due to the complex and dynamic regulation of body fluid balance [16] and the lack of clear criteria for dehydration [27,38].

### 4.1. The Impact of the Time of Day: Time Points (before or after Work)

Indices of hydration status may change depending on the time of day (time of collection of biological material), especially those concerning urine [39]. It has been shown that urine concentration varies depending on the time of day [39]. Morning urine (both immediately after waking up and later) is more concentrated than that produced in the early and late afternoon, and 24 h urine (most likely because of overnight fluid break and decreasing of glomerular filtration during sleep) [16,39]. The lowest levels of U_osm_, USG, and U_col_ were observed in the late afternoon. In turn, the peak of their concentrations was observed in urine collected after the night break (first morning urine) and in subsequent samples collected in the morning [39]. Moreover, changes in fluid intake may impact urine concentration and urine volume, which stabilize within 24 h of modifying fluid volume intake [39]. 

In the study of foresters, no impact of the time of day was observed for indices such as urine osmolality, serum osmolality, urine color, percentage of body mass loss, and percentage of reduction in total body water. However, a high percentage of foresters started working improperly hydrated (including severe dehydration).

In the case of specific urine gravity, higher values of USG in the morning before work on the first day and on the following day (time points 1 and 3, respectively; first morning urine) were noted compared to USG after work. Contrary to this study, some studies have found higher USG values at the end of work compared to baseline in forestry workers [14,22,25] and construction workers [28], which may be indicative of a deteriorated hydration status during work. Discrepancies between these reports and our study may result, among others, from differences in air temperatures at which the work was performed, types of work (metabolic rate, sweating), shift duration, or the time of urine collection (before or after eating and drinking). It should be emphasized that in the present study, the time of fluid and food intake at work was not controlled, which may have influenced the results. 

It is worth noting that there are no clear criteria for urine-specific gravity levels that would indicate workers’ dehydration [27,38], but the value <1.020 is mostly used as a cut-off point of euhydration (normal total body water) [12,16,25,40,41]. In the present study, when this criterion was applied, 65%, 50%, and 52% of foresters were considered dehydrated before work on the first day, on the following day, and after work, respectively. This indicated that more than half of the foresters arrived to work with some dehydration. Other studies have also shown that a high percentage of outdoor workers were dehydrated at the beginning of their work [14,25,26,42]. In a study of forestry workers harvesting trees in South Africa, Biggs et al. [25] showed that 44% were dehydrated on arrival at work. Inadequate hydration before work was observed also among loggers [14,22]. It should be emphasized that even slight dehydration may affect, for example, the mechanisms of thermoregulation and mental and physical abilities to perform work [10,11,12]. 

Not only insufficient fluid intake can be hazardous to health but also drinking too much [43]. This study showed (using the criteria of Biggs et al. [25]) that after work, 13% of foresters were overhydrated (USG < 1.012), which may suggest inadequate fluid intake. Similarly, several forestry workers from South Africa were overhydrated both after the shift and before work [25]. It was suggested that excessive hypotonic fluid intake, along with limiting the amount of salt in the diet, might contribute to hyponatremia and hyperhydration [25]. According to the recommendations of Kenefick [44], workers should not drink too much fluid as it would result in weight gain. That is why appropriate education programs regarding the amount and type of fluids consumed while working in various environmental conditions are so important. 

The changes in body mass were not a good indicator of the hydration status after work in foresters, as during the shift, they consumed both fluids and food, which is consistent with a study by Biggs et al. [25]. It was suggested that the morning body mass after an overnight break/fasting should be monitored to control body mass as a marker of dehydration [11,18]. In the present study, no body mass loss of more than 1% was observed after work compared to that before work. Contrary to the results, other studies of forestry workers found changes in body mass of over 2% [25]. Increments in both body mass and total body water after work observed in this study may suggest that workers were not consuming sufficient amounts of fluids and food to meet their needs.

### 4.2. The Impact of Season of the Year on Hydration Status

Our study of foresters found the influence of the season of the year on the indices of the hydration status. Higher serum osmolality levels were recorded in autumn and winter than in the summer period. Moreover, in winter, the percentage of dehydrated workers with S_osm_ ≥290 mOsmol/kg H_2_O was significantly higher in all measured points than in summer. On the other hand, the study did not find the influence of the season of the year on urine-specific gravity, which is consistent with the findings of previous studies [14,25,30]. The lack of differences was most likely due to the widespread dehydration (defined as any value ≥1.020 g/mL) among foresters at any season of the year, which was also confirmed by the research of forestry workers from New Zealand and South Africa [14,25]. Biggs et al. [25] observed that 43% and 47% of forestry workers in autumn and winter, respectively, were dehydrated before work. A high percentage of dehydrated workers in low temperatures may result from using incorrect protective clothing and/or modification of fluid intake (reduction in fluid volume) due to the belief that, at lower temperatures, there is no risk of dehydration [14]. These results again confirm the need for education on factors that may affect fluid requirements and how to prevent dehydration.

When studying the effect of the time of day and season of the year on urine indices of hydration status, it is important to note that urine concentration (osmolality, specific gravity, color) is influenced by many factors, e.g., fluid intake, diet, exercise, ambient temperature, and the time from the previous voiding [41,45]. It should also be stressed that urinary indices of hydration status reflect the renal response to fluid homeostasis (i.e., maintenance of osmolality and plasma volume) and do not reflect real-time hydration status at a cellular level with regard to osmoregulation and volume status [41,46]. Therefore, it is suggested that, based on urine hydration markers, the most accurate assessment of hydration status can be made by examining hydration indices in the first morning urine [45]. In the present study, pre-work tests were performed in a fasting state to eliminate potential factors that could affect the results. However, after work, participants were asked to refrain from voiding for 2 h before sample collection. Due to the fact that the authors wanted to reflect the typical workday, they did not interfere with the amount, time, and type of fluids consumed by foresters. The workers consumed the last fluids at work at least 30 min before the end of work and the collection of biological material. Therefore, it should be noted that the results obtained after work may not exactly reflect water balance but rather show acute changes in total body water [45]. It should also be emphasized that the presented results were obtained from monitoring a small number of workers of one gender and profession, and therefore, they should be interpreted with caution.

### 4.3. The Effect of Selected Hydration Status Markers on the Prevalence of Dehydration

In this study, urine indices of hydration status did not correlate with the blood hydration status markers, which is consistent with the results of previous studies [47,48,49]. Based on serum osmolality, adequate hydration was maintained in 83% of foresters before work on the following day. Dehydration was found in 55% of workers based on urine osmolality ≥700 mOsmol/kgH_2_O and 50% of workers based on USG ≥1.020. Raines et al. [26] demonstrated that the percentage of discordant classifications of dehydration before the shift was 20% for U_col_ and plasma osmolality indices. It was suggested that weak correlations or lack of relationships between urine and blood hydration indices might be due to a delayed response to acute changes in hydration status measured in blood and urine [48]. On the other hand, significant correlations were observed between urine indices of hydration such as urine color, urine-specific gravity, and urine osmolality, which has been confirmed by others [28,47,49]. 

It is worth noting that the percentage of workers identified as dehydrated was similar for the morning tests of thirst, urine color, urine-specific gravity, and urine osmolality. This may suggest that relying on these indices will allow for the identification of workers dehydrated before work. This is in line with recommendations [11,18,45] for workers to monitor morning body mass, urine color, and thirst. Then, if a worker meets at least two of the dehydration criteria, he or she is most likely dehydrated [18,19]. 

To reduce the risk of dehydration in workers, it is important to implement education programs concerning adequate fluid intake at work and at home [11,24,29,50]. It was shown that rigid rules concerning fluid intake that are the same for all individuals might not prevent dehydration in workers [51]. Consequently, more attention should now be paid to meeting individual fluid requirements contained in tailored hydration plans [52,53]. However, to achieve this, workers should know how to monitor their hydration status. According to recommendations, dehydration is best diagnosed by clinical observations based on a combination of medical history, physical examination, laboratory results, and clinical experience [38]. However, in the case of workers, it is difficult to perform laboratory tests on a daily basis. Therefore, it is recommended to monitor body mass, urine color, thirst [11], and voiding frequency [54].

There were several limitations of this study. As in other pilot studies, the small sample size limits the statistical power and does not allow for drawing definitive conclusions. Additionally, the fact that the study participants were volunteers is a potential source of bias. The research was only conducted at three time points. Moreover, most of the workers had a BMI above 25, which may also have influenced the results. Despite the limitations, the study also has strengths. The participants were of one gender and the same profession, which reduced the effect of external factors (e.g., work task) on the indices studied. The study was conducted at different seasons of the year, which made it possible to compare the effect of ambient temperature on hydration status. Hydration status was assessed using various indices—both subjective and objective—assessed in both urine and blood. 

## 5. Conclusions

Hydration status indices were influenced by both the season of the year (ambient temperature) and the time of day. In addition, dehydration was found in many workers at the beginning of work, which can affect their health, ability to work, and safety at work. The high prevalence of dehydration in workers indicates the need for the implementation of educational programs to ensure optimal hydration. Workers should know how to monitor their hydration status (e.g., by checking urine color or feeling thirsty) and their fluid intake (e.g., by body mass measurements before and after work). In addition, they should be aware of the symptoms of dehydration, both early (e.g., decreased urine output, thirst, dry mouth, headache) and late (acute) (e.g., feeling unusually tired, extremely thirsty, little or no voiding, changed urine color, loss of skin elasticity, low blood pressure, rapid heartbeat, and accelerated breathing). 

## Figures and Tables

**Table 1:** Criteria for classification of a dehydrated state [12,18,19,36,37].

Hydration Indices	Cut-off Points for Dehydration
Thirst [18]	Answer “yes”
Urine color [19,37]	5–8
Urine osmolality [12,19]	≥700 mOsmol/kg H_2_O
Urine-specific gravity [12,19]	≥1.020 g/ml
Serum osmolality [12,19]	≥290 mOsmol/kg H_2_O
Body mass (percentage change) [12,19]	≥1%
Total body water (percentage change) [12]	≥2%
Serum osmolality (percentage change) [36]	≥2%

**Table 2:** The characteristics of the study participants.

	Summer (*n* = 20)	Autumn (*n* = 21)	Winter (*n* = 19)	All (*n* = 60)
Age (years)	30.2 ± 7.9	33.0 ± 6.2	33.9 ± 5.9	32.3 ± 6.8
Body mass (kg)	84.9 ± 14.0	93.3 ± 19.2	91.5 ± 10.6	89.9 ± 15.4
Body height (cm)	177.0 ± 7.3	179.8 ± 4.9	181.7 ± 6.3	179.5 ± 6.4
BMI (kg/m^2^)	27.1 ± 4.5	28.7 ± 5.1	27.7 ± 3.1	27.9 ± 4.3
Body fat percentage	23.9 ± 6.6	25.1 ± 6.7	22.0 ± 5.4	23.7 ± 6.3

**Table 3:** The results of the assessment of hydration status indices at time points: mean ± standard deviation and the number (percentage) of dehydrated workers (where applicable).

Hydration Status Indices	Time Point		
	1—Before Work (Baseline)	2—After Work	3—Before Work on the Following Day
Thirst	- 28 (47)	- 21 (35)	- 29 (48)
Body mass (kg)	89.9 ± 15.4 -	90.2 ± 15.4 * -	90.0 ± 15.3 ^#^ -
Total body water (L)	49.8 ± 6.2 -	50.2 ± 6.2 * -	50.0 ± 6.1 * -
Urine color	4 ± 2 25 (42)	4 ± 1 18 (30)	4 ± 1 26 (43)
Urine-specific gravity (g/mL)	1.022 ± 0.006 39 (65)	1.019 ± 0.006 * 31 (52)	1.020 ± 0.006 ^#^ 30 (50)
Urine osmolality (mOsmol/kg H_2_O)	760 ± 217 39 (65)	736 ± 213 37 (62)	724 ± 219 32 (53)
Serum osmolality (mOsmol/kg H_2_O)	283 ± 6 7 (12)	285 ± 5 7 (12)	286 ± 6 10 (7)

* Significantly (*p* < 0.05) different from time point 1 (baseline); ^#^ Significantly (*p* < 0.05) different from time point 2 (after work).

**Table 4:** Hydration status indices depending on the season of the year: mean ± standard deviation and the number (percentage) of dehydrated workers (where applicable).

Hydration Status Indices	Summer			Autumn			Winter		
	1—Before Work (Baseline)	2—After Work	3—Before Work on the Following Day	1—Before Work (Baseline)	2—After Work	3—Before Work on the Following Day	1—Before Work (Baseline)	2—After Work	3—Before Work on the Following Day
Thirst	- 6 (30)	- 6 (30)	- 7 (35)	- 12 (57)	- 8 (38)	- 9 (43)	- 10 (53)	- 7 (37)	- 13 (68)
Body mass (kg)	84.9 ± 14.0 -	85.0 ± 13.8 -	84.9 ± 13.8 -	93.3 ± 19.2 -	93.7 ± 19.4 -	93.4 ± 19.3 -	91.5 ± 10.6 -	92.0 ± 10.5 -	91.6 ± 10.4 -
Total body water (L)	46.8 ± 5.5 -	47.3 ± 5.4 -	47.1 ± 5.1 -	50.5 ± 7.3 -	50.9 ± 7.5 -	50.8 ± 7.5 -	52.0 ± 4.2 * -	52.4 ± 4.2 -	52.1 ± 4.1 -
Urine color	5 ± 1 8 (40)	4 ± 1 8 (40)	5 ± 1 11 (55)	4 ± 1 9 (43)	4 ± 1 5 (24)	4 ± 1 6 (29)	4 ± 2 8 (42)	3 ± 1 5 (26)	4 ± 1 9 (47)
Urine specific gravity (g/mL)	1.022 ± 0.006 14 (70)	1.019 ± 0.006 9 (45)	1.021 ± 0.004 10 (50)	1.021 ± 0.007 12 (57)	1.018 ± 0.005 10 (48)	1.019 ± 0.007 9 (49)	1.022 ± 0.006 13 (68)	1.020 ± 0.006 12 (63)	1.021 ± 0.007 11 (42)
Urine osmolality (mOsmol/kg H_2_O)	831 ± 184 15 (75)	732 ± 233 11 (55)	734 ± 174 10 (50)	696 ± 244 11 (52)	731 ± 210 13 (62)	711 ± 264 11 (52)	756 ± 206 13 (68)	756 ± 206 13 (68)	756 ± 206 11 (58)
Serum osmolality (mOsmol/kg H_2_O)	282 ± 2 0 (0)	282 ± 3 0 (0)	283 ± 3 0 (0)	284 ± 4 1 (5)	286 ± 5 1 (5)	287 ± 6 * 5 (24) *	284 ± 10 6 (32) *^#^	288 ± 6 * 6 (32) *^#^	287 ± 9 5 (26) *

* Significantly (*p* < 0.05) different from time point in the Summer, ^#^ Significantly (*p* < 0.05) different from time point in the Autumn.

**Table 5:** Percentage of dehydrated participants depending on the hydration status indices.

Hydration Status Indices	Index No.	Percentage of Dehydrated Participants (Time Point 2)	Percentage of Dehydrated Participants (Time Point 3)
Percentage change of body mass **^a^**	1	0.0%	5.0%
Urine color	2	30.0% ^1,4,7^	43.3% ^1,4,7^
Thirst	3	35.0% ^1,4,7^	48.3% ^1,4,7,8^
Percentage change of total body water **^a^**	4	1.7%	3.3%
Urine-specific gravity	5	51.7% ^1,2,4,7,8^	50.0% ^1,4,7,8^
Urine osmolality	6	61.7% ^1,2,3,4,7,8^	53.3% ^1,4,7,8^
Serum osmolality	7	11.7% ^1,4^	16.7% ^1,4^
Percentage change of serum osmolality **^a^**	8	20.0% ^1,4^	27.0% ^1,4^

**^a^** change compared to time point 1 (baseline value); * Superscript numbers denote significant differences (*p* < 0.05); subsequent digits correspond to the respective variables marked in column Index No.

## Data Availability

Not applicable.
